# Tumor budding and lymphovascular invasion as prognostic factors in p16-positive oropharyngeal squamous cell carcinomas

**DOI:** 10.1038/s41416-024-02912-3

**Published:** 2024-11-29

**Authors:** Fabian Stögbauer, Markus Wirth, Maren Lauterbach, Barbara Wollenberg, Benedikt Schmidl, Cosima C. Hoch, Iordanis Ourailidis, Jochen Hess, Markus Eckstein, Arndt Hartmann, Heinrich Iro, Antoniu-Oreste Gostian, Matthias Balk, Moritz Jesinghaus, Julika Ribbat-Idel, Verena-Wilbeth Sailer, Sven Perner, Karl-Ludwig Bruchhage, Markus Hoffmann, Lukas Lükewille, Christiane Maria Stuhlmann-Laeisz, Christoph Röcken, Carolin Mogler, Jan Budczies, Melanie Boxberg

**Affiliations:** 1 Technical University of Munich, TUM School of Medicine and Health, Institute of General and Surgical Pathology, Munich, Germany; 2 Department of Otolaryngology, Head and Neck Surgery, School of Medicine and Health, Technical University of Munich (TUM), Munich, Germany; 3 University of Heidelberg, Institute of Pathology, Heidelberg University Hospital, Heidelberg, Germany; 4 Department of Otorhinolaryngology, Head and Neck Surgery, Experimental Head and Neck Oncology, Heidelberg University Hospital, Heidelberg, Germany; 5 Institute of Pathology, University Hospital Erlangen, Friedrich-Alexander-Universität Erlangen-Nürnberg, Erlangen, Germany; 6 Bavarian Cancer Research Center (Bayerisches Zentrum für Krebsforschung, BZKF), Erlangen, Germany; 7 Comprehensive Cancer Center EMN, University Hospital Erlangen, Friedrich- Alexander- Universität Erlangen- Nürnberg, Erlangen, Germany; 8 Department of Otolaryngology, Head & Neck Surgery, University Hospital Erlangen, Friedrich-Alexander-Universität Erlangen-Nürnberg, Erlangen, Germany; 9 Department of Otorhinolaryngology, Merciful Brothers Hospital St. Elisabeth, Straubing, Germany; 10 Institute of Pathology, University Hospital Marburg, Marburg, Germany; 11 Institute of Pathology, University of Lübeck, Lübeck, Germany; 12 Pathology of the University Hospital Schleswig-Holstein, Campus Luebeck, Luebeck, Germany; 13 Pathology, Research Center Borstel, Leibniz Lung Center, Borstel, Germany; 14 Center for Precision Oncology Tübingen, Tübingen, Germany; 15 Department of Otorhinolaryngology, University of Luebeck, Luebeck, Germany; 16 Department of Otorhinolaryngology, Head and Neck Surgery, Christian-Albrechts-University Kiel, Kiel, Germany; 17 Department for Pathology, Christian-Albrechts-University of Kiel, Kiel, Germany

**Keywords:** Prognostic markers, Head and neck cancer, Surgical oncology

## Abstract

**Background:**

We aimed to validate the prognostic significance of tumor budding (TB) in p16-positive oropharyngeal squamous cell carcinomas (OPSCC).

**Methods:**

We analyzed digitized H&E-stained slides from a multicenter cohort of five large university centers consisting of *n* = 275 cases of p16-positive OPSCC. We evaluated TB along with other histological parameters (morphology, tumor-stroma-ratio, lymphovascular invasion (LVI), perineural invasion) and calculated survival outcomes using both univariate and multivariate analyses.

**Results:**

TB was identified as an independent prognostic parameter, with TB-high cases showing inferior outcomes in univariate (HR: 3.08, 95%-CI: 1.71–5.54) and multivariate analyses (HR: 4.03, 95%-CI: 1.65–9.83). Similarly, LVI remained an independent prognostic factor (HR: 3.00, 95%-CI: 1.22–7.38). A combined classification including TB and LVI stratified cases into low-, intermediate- and high-risk categories. We could not detect correlations between TB and the number of lymph node metastases or between TB and an extracapsular extension of lymph node metastases.

**Conclusions:**

In addition to LVI, we could identify TB as an independent prognostic factor in p16-positive OPSCC in this multicenter study cohort. Thus, evaluating TB along with LVI in a combined scheme for prognostication might help to establish a more personalized treatment regimen for patients with p16-positive OPSCC.

## Background

Approximately 70% of oropharyngeal squamous cell carcinomas (OPSCC) are caused by infections with human papillomaviruses (HPV) [1]. In clinical routine, the expression of p16 serves as a surrogate parameter with high sensitivity and specificity for HPV [2, 3]. Studies have demonstrated that p16-positive OPSCC exhibit superior survival rates and better responses to radiotherapy compared to p16-negative OPSCC [4–6]. Consequently, the latest World Health Organization (WHO) classification for head and neck tumors and the 8th edition of the TNM Classification of Malignant Tumors distinguish p16-positive OPSCC as a separate disease entity [3, 7, 8]. Comprehensive genomic profiling of large head and neck cancer cohorts supports the distinct tumor biology of HPV-positive versus HPV-negative subtypes [9]. Given the comparatively favorable prognosis of p16-positive OPSCC and the severe side effects associated with radiotherapy, treatment de-escalation strategies of therapeutic regimens are under investigation [10, 11]. However, attempts to validate these strategies have not been successful to date [12]. It is worth noting that approximately 20% of HPV-positive OPSCC cases elicit poor outcomes, and currently, no biomarkers have been established to identify these high-risk cases [13].

In previous studies, our group and others validated tumor budding (TB) as a prognostic biomarker in HPV-negative head and neck squamous cell carcinomas (HNSCC) [14, 15]. High TB was associated with inferior disease-free and overall survival (OS) as well as increased metastatic spread to lymph nodes [16]. In contrast, studies on the prognostic implications of TB in HPV-positive tumors are scarce [17].

In a more recent analysis of the TCGA HNSCC cohort, we identified TB as a negative prognostic biomarker not only in HPV-negative tumors but also in the HPV-positive subgroup [18]. Another potential prognostic factor in HNSCC comprises the evaluation of the tumor-stroma-ratio (TSR) which seems to be of prognostic significance in head and neck cancer including tumors of the oropharynx [19, 20]. The prognostic significance of the TSR was especially shown for cancers of the oral cavity where stroma-rich tumors are associated with a reduced disease-specific and disease-free survival [19]. However, in a meta-analysis Almangush et al. detected some degree of heterogeneity in the determination of the disease-specific survival [19]. Furthermore, the prognostic significance of the TSR in other head and neck subsites (e.g., of the nasopharynx or larynx) is unclear due to a limited number of studies published so far [21].

Furthermore, evaluating tumor-infiltrating lymphocytes (TILs) might help in the prognostic stratification of OPSCC as high amounts of TILs are linked to improved survival outcomes in these tumors [22].

In our study, we aimed to validate the prognostic significance of TB and to probe the prognostic impact of other morphological parameters (TSR, TILs) in a large multicenter cohort of p16-positive HPV-associated OPSCC. We included *n* = 275 p16-positive OPSCC from five pathology departments in Germany (Friedrich-Alexander University Erlangen-Nuremberg, University of Heidelberg, University of Lübeck, Christian-Albrechts-University Kiel, Technical University of Munich), rendering the cohort the up to date largest cohort analyzing TB in HPV-associated OPSCC. Our retrospective study contributes to the identification of biomarkers that could guide therapy and, specifically, aid in selecting patients for treatment de-escalation in HPV-related OPSCC.

## Material and methods

### Patient cohort

Included in the study cohort were resection specimens of OPSCC whereas biopsies were not considered applicable. The initial study cohort consisted of 299 patients with p16-positive squamous cell carcinomas originating in the oropharynx. A total of *n* = 24 had to be excluded from the histomorphological analysis as e.g. too less invasive tumor for the evaluation of tumor budding was contained on the digitized slides or the slides contained a lymph node metastasis of the resection specimen and not the primary tumor.

The final study cohort consisted solely of resection specimens of *n* = 275 primary, treatment-naive OPSCC. Cases were enrolled from five pathology departments in Germany - the Friedrich-Alexander University Erlangen-Nuremberg, University of Heidelberg, University of Lübeck, Christian-Albrechts-University Kiel and the Technical University of Munich - rendering the study design a retrospective multicenter study. Resections were performed between 1991 and 2023 (median 2015, interquartile range 5 years). For routine diagnostic pathology purposes, formalin-fixed, paraffin embedded (FFPE) tumor tissue blocks and subsequently Hematoxylin-Eosin (H&E) stains were created. For our study, OPSCC patients were identified in pathology archives and were included in a consecutive manner. As p16-positivity is a widely accepted surrogate marker for HPV-association in case of HNSCC located within lymphoepithelial regions within the oropharynx [3, 23, 24], p16-staining was performed in each center individually and after evaluation of the stain, patients were enrolled based on presence of p16-positivity with p16-negative OPSCC counterparts being excluded.

**Fig. 1:**
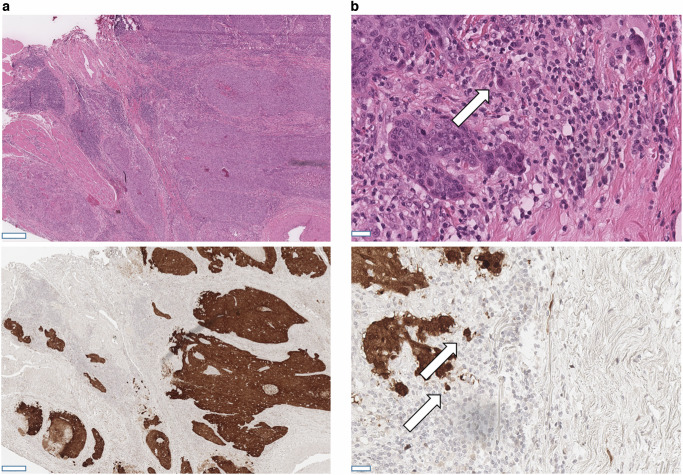
Microscopic illustration of a case with basaloid appearance and strong diffuse p16 expression. **a** Overview over a OPSCC in low magnification; upper image: H&E, lower image: p16 immunohistochemistry; scalebar in both images: 200 µm. **b** High magnification view of selected areas with tumor buds highlighted by arrows. (upper image: H&E, lower image: p16 immunohistochemistry; scalebar in bith images: 20 µm). OPSCC: oropharyngeal squamous cell carcinoma; H&E: Hematoxylin-Eosin stain.

In accordance with the guideline from the College of American Pathologists, moderate to strong p16-expression in at least 70% of tumor cells was regarded as positive (Fig. 1; [23]). The analysis of immunohistochemical p16 stains was performed locally in each university pathology department and evaluated on glass slides by board-certified pathologists with long experience in head and neck pathology named including affiliations in our author list.

Median follow-up periods were 48 months for OS and 44.3 months for progression-free survival (PFS). To facilitate the comparability of cases all cases were staged according to the 4th edition of the “WHO classification of Head and Neck Tumors” [3]. Detailed clinicopathological characteristics of the study cohort, which were collected separately in each university center in a joint clinico-pathological effort by accordingly qualified medical doctors as named including affiliations in our author list, are given in Supplementary Tables 1 and 2.

### Histological analyses

For histological analyses, routine H&E stained tumor sections were digitized by scanning with 40x magnification and saved as *.svs file. One digitized H&E-stained slide per case with unambiguous squamous cell carcinoma morphology was provided by all five participating centers and was evaluated by two experienced pathologists (FS and MB) blinded to clinicopathological data. Cases were subtyped in analogy to the WHO classification for p16-negative HNSCC in conventional (keratinizing vs. non-keratinizing) and basaloid morphology (Fig. 1) [3]. In analogy with previous studies in HNSCC, TB was defined as the detachment of four or less tumor cells from the main tumor mass, which infiltrated the adjacent stroma [18, 25]. TB was counted irrespective of the location within the tumor, i.e., tumor center or periphery. We analyzed TB in 1 high-power field (HPF) with the highest amount of buds (“hotspot”) and ten consecutive HPF with 1 HPF covering an area of 97464 µm^2^ and corresponding to a field diameter of 0.35 mm in light microscopy.

The stroma content (evaluated as TSR) was assessed as the proportion of the tumor area covered by stroma with regard to the whole tumor area. Areas of necrosis were excluded from the assessment.

TILs evaluation was based on the assessment of stromal TILs. The stromal area covered by TILs (under the exclusion of intraepithelial TILs) was evaluated in relation to the whole tumor stroma [26].

### Statistical analyses

For the morphological parameters TB, stroma content and immune infiltrate, “Cutoff Finder” was applied to identify the prognostically most relevant cutoffs [27].

Ordinally scaled data were compared with Fisher’s exact test, whereas continuous data were compared with the Mann–Whitney-U-Test. Mutual correlations were calculated with Spearman’s correlation coefficient. Survival outcomes between groups were analyzed with the Kaplan–Meier method and survival differences were calculated with the Log-Rank test. For univariate and multivariate survival analysis a Cox Regression model was applied.

All tests were calculated two-sided with *p*-values < 0.05 regarded as significant. For statistical analyses R (version 4.1.3) was used.

## Results

### Histological analyses

Detailed clinicopathological characteristics of the *n* = 275 patients comprising the study cohort are shown in Supplemental Table 1. All tumors were located in the oropharynx, with *n* = 95 (34.5%) originating within the tonsils, *n* = 17 (6.2%) originating from the base of the tongue, *n* = 2 (0.7%) originating from the lateral oropharyngeal wall, *n* = 1 (0.4%) originating from the soft palate and the remaining *n* = 160 (58.2%) were not assigned to a specific anatomic subsite.

Basic characteristics for TB (mean, median, minimum, maximum) in 1 HPF, TB in 10 HPF, stroma content and TILs are shown in Supplementary Table 2. A total of *n* = 134 tumors (48.7%) showed no tumor budding at all, whereas in *n* = 141 cases (51.3%) TB was observed in varying degrees.

To determine the cutoffs for each biomarker with the strongest prognostic impact on OS we applied an online tool for biomarker cutoff optimization “Cutoff Finder”, described in detail in ref. [27]. The optimal cutpoints to separate low-risk from high-risk cases were those with the lowest *p*-values in a subsequent log-rank test. The following cutpoints for high-risk cases for poor outcome were calculated: For TB in 1 HPF OPSCC with ≥4 buds showed significantly worse OS compared to counterparts with <4 buds, for TB in 10 HPF as well ≥4 buds provided an optimal discrimination and for TILs cases with ≥53% showed a significantly better OS. For the TSR no prognostically significant cutoff was identified.

All tested cutoffs for TB (ranging from 1 to 12) yielded a prognostically significant stratification (Supplementary Fig. 1).

As both the optimal cutoff value (4 buds) and the applied cutoff value (6 buds) showed similar hazard ratios we classified the cases according to TB in 10 HPF and applied the cutoffs identified in our previous publication on HNSCC to facilitate the comparability with our previous study [18]. To this end, high TB was defined as detection of at least 6 buds for the 10 HPF methods and as detection of at least 2 buds for the 1 HPF method. For high TILs the cutoff ≥53% was applied.

### Clinicopathologic correlations

65 cases (23.6%) were classified as TB-high and 93 cases (33.8%) as TILs-high.

TB evaluated in 1 HPF and TB evaluated in 10 HPF were strongly correlated (Spearman’s rho = 0.98, *p* < 0.001). Analyzing 10 HPF cases with high TB were less often classified as pT1/2 and more often classified as UICC stage III/IV.

Neither TB in 1 HPF (Spearman’s rho = 0.14, *p* = 0.054) nor in 10 HPF (Spearman’s rho = 0.13, *p* = 0.068) were associated with the number of positive lymph nodes nor with the pN0/pN+ stage. For *n* = 43 patients, information regarding an extracapsular extension of a lymph node metastasis was available but we could not detect an association with TB.

Cases with high TILs were more often classified as pT1 or pT2, more often pN0 and more often UICC stage I/II.

The number of buds in 1/10 HPF showed a weak negative correlation with the amount of TILs (Spearman’s rho: −0.144/−0.137, *p* = 0.017/0.023) and a weak positive correlation with the stroma content (Spearman’s rho: 0.214/0.207, *p* < 0.001 each). No correlation between the amount of TILs and the stroma content was detected.

Clinicopathological correlations are summarized in Table 1.

**Table 1:** Clinicopathological characteristics of the cohort after stratification into TB low and TB high according to the cutoff for 10 HPF.

	TB low	TB high		TILs low	TILs high	
	*n* (%)	*n* (%)	*p*	*n* (%)	*n* (%)	*p*
Age	median 61 (IQR 12)	median 62 (IQR 14)	0.979	median 61.5 (IQR 13)	median 61 (IQR 10)	0.267
Sex			0.536			
male	149 (71.0)	43 (66.2)		128 (70.3)	64 (68.8)	0.890
female	61 (29.0)	22 (33.8)		54 (29.7)	29 (31.2)	
pT			**0.036**			**<0.001**
pT1	57 (28.5)	21 (35.0)		38 (22.5)	40 (44.0)	
pT2	113 (56.5)	23 (38.3)		88 (52.1)	48 (52.7)	
pT3	24 (12.0)	11 (18.3)		33 (19.5)	2 (2.2)	
pT4	6 (3.0)	5 (8.3)		10 (5.9)	1 (1.1)	
N/A	15					
pN			0.713			**0.009**
pN0	41 (21.2)	13 (23.6)		120 (73.2)	74 (88.1)	
pN+	152 (78.8)	42 (76.4)		44 (26.8)	10 (11.9)	
N/A	27					
UICC stage			**0.013**			**0.004**
I/II	37 (18.7)	11 (18.6)		38 (22.8)	10 (11.1)	
III	99 (50.0)	18 (30.5)		64 (38.3)	53 (58.9)	
IV	62 (31.3)	30 (50.8)		65 (38.9)	27 (30.0)	
N/A	18					
Resection status			0.741			0.057
R0	123 (78.3)	37 (74.0)		96 (77.4)	64 (77.1)	
R1/R2	22 (14.0)	8 (16.0)		14 (11.3)	16 (19.3)	
RX	12 (7.6)	5 (10.0)		14 (11.3)	3 (3.6)	
N/A	68					
Extracapsular extension			0.575			0.483
absent	23 (57.5)	1 (33.3)		17 (32.1)	2 (18.2)	
present	17 (42.5)	2 (66.7)		36 (67.9)	9 (81.8)	
N/A	232					
Lymphovascular invasion			0.718			0.328
absent	172 (81.9)	52 (80.0)		145 (79.7)	79 (84.9)	
present	38 (18.1)	13 (20.0)		37 (20.3)	14 (15.1)	
Perineural invasion			0.286			0.341
absent	186 (88.6)	54 (83.1)		156 (85.7)	84 (90.3)	
present	24 (11.4)	11 (16.9)		26 (14.3)	9 (9.7)	
Morphology			0.068			0.266
basaloid	70 (33.3)	12 (18.5)		56 (30.8)	26 (28.0)	
conventional (keratinizing)	57 (27.1)	21 (32.3)		56 (30.8)	22 (23.7)	
conventional (non-keratinizing)	83 (39.5)	32 (49.2)		70 (38.5)	45 (48.4)	
Center			**0.017**			**<0.001**
Erlangen	83 (39.5)	40 (61.5)		56 (30.8)	67 (72.0)	
Heidelberg	27 (12.9)	9 (13.8)		28 (15.4)	8 (8.6)	
Kiel	10 (4.8)	1 (1.5)		3 (1.6)	8 (8.6)	
Lübeck	45 (21.4)	9 (13.8)		46 (25.3)	8 (8.6)	
Munich	45 (21.4)	6 (9.2)		49 (26.9)	2 (2.2)	

*IQR* Interquartile range, *N/A* Not available, *TB* Tumor budding, *TILs* Tumor infiltrating lymphocytes.

Bold values indicate statistical significance.

### Survival analysis

In univariate survival analysis (OS) age, UICC stage IV, R1/R2-resection status, LVI and TB were associated with inferior outcomes while high levels of TILs were associated with a lower hazard ratio (Table 2, Supplementary Figs. 2, 3). Cases with high TB and LVI were also associated with inferior survival outcomes regarding PFS (Supplementary Table 3, Supplementary Figs. 2, 3). Median survival times for OS (PFS) for high TB were 116.0 (138.0) months. Median survival times for OS for absence of LVI were 256.0 months and 259.0 months for presence of LVI. For low TB (OS, PFS) and LVI (PFS) no median survival times could be calculated as the survival probability remained >0.5.

**Table 2:** Results for the univariate analysis are shown for TB assessment in 10 HPF and for overall survival.

		HR	HR (95% CI)	*p*
Age		1.05	1.01–1.08	**0.004**
Year of surgery		0.99	0.95–1.04	0.772
Sex				
	male	1		
	female	0.73	0.38–1.39	0.337
UICC stage				
	stage I/II	1		
	stage III	1.52	0.43–5.36	0.513
	stage IV	4.25	1.28–14.14	**0.018**
Resection status				
	R0	1		
	R1/R2	3.15	1.29–7.68	**0.012**
	RX	2.14	0.70–6.56	0.185
Lymphovascular invasion				
	absent	1		
	present	2.04	1.09–3.82	**0.027**
Perineural invasion				
	absent	1		
	present	1.91	0.91–3.97	0.085
Morphology				
	basaloid	1		
	keratinizing	2.26	0.94–5.42	0.068
	non-keratinizing	1.88	0.80–4.42	0.150
Center				
	Erlangen	1		
	Heidelberg	0.95	0.41–2.20	0.908
	Kiel	0	0-Inf	0.996
	Lübeck	1.65	0.85–3.20	0.136
	Munich	0.47	0.14–1.58	0.221
TIL				
	low	1		
	high	0.26	0.10–0.66	**0.005**
Tumor budding				
	low	1		
	high	3.08	1.71–5.54	**<0.001**

*HR* Hazard ratio, *95% CI* 95% confidence interval, *TIL* Tumor infiltrating lymphocyte, *UICC* Union internationale contre le cancer.

Bold values indicate statistical significance.

Both the year of surgical treatment and the center of clinical care were not associated with survival outcomes.

In multivariate analysis age and sex were included along with the significant parameters of the univariate analysis (UICC stage, resection status, LVI, TILs, TB). For OS age, LVI and TB remained as independent prognostic factors (Table 3) whereas for PFS only LVI and TB remained as independent prognostic factors (Supplementary Table 4).

**Table 3:** Results for the multivariate analysis are shown for TB assessment in 10 HPF and for overall survival.

		HR (mean)	HR (95% CI)	*p*
Age		1.06	1.01–1.11	**0.011**
Sex				
	Male	1		
	Female	0.9	0.34–2.42	0.841
UICC stage				
	Stage I/II	1		
	Stage III	1.94	0.22–17.01	0.551
	Stage IV	3.63	0.42–31.15	0.240
Resection status				
	R0	1		
	R1/R2	2.12	0.74–6.08	0.162
	RX	0.67	0.16–2.72	0.571
Lymphovascular invasion				
	Absent	1		
	Present	3.00	1.22–7.38	**0.016**
TILs				
	Low	1		
	High	0.46	0.15–1.45	0.186
Tumor budding				
	Low	1		
	High	4.03	1.65–9.83	**0.002**

*HR* Hazard ratio, *95% CI* 95% confidence interval, *TILs* Tumor infiltrating lymphocytes, *UICC* Union internationale contre le cancer.

Bold values indicate statistical significance.

### Combined classification of TB and LVI

Applying the combined prognostic stratification scheme composed of TB in 10 HPF and LVI we obtained three subgroups: a) Cases with low TB and absence of LVI (*n* = 172, 62.5%), b) cases with low TB and presence of LVI or cases with high TB and absence of LVI (*n* = 90, 32.7%) and c) cases with high TB and presence of LVI (*n* = 13, 4.7%).

**Fig. 2:**
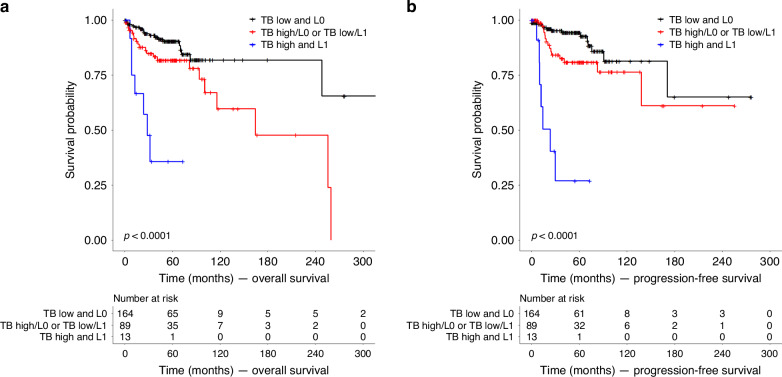
Kaplan–Meier plots for illustration of survival of patients depending on TB and LVI status. **a** Overall survival (**b**) Progression free survival. Patients were stratified according to TB in 10 HPF and the absence or presence of LVI. HPF High-power field, L0 Lymphovascular invasion absent, L1 Lymphovascular invasion present, LVI Lymphovascular invasion, TB Tumor budding.

In univariate analysis (OS) the combined prognostic stratification scheme yielded an intermediate risk for inferior outcomes for cases with either high TB or presence of LVI (HR: 2.18, 95% CI: 1.16–4.11, *p* = 0.016) and the highest risk for cases with both high TB and the presence of LVI (HR: 9.78, 95% CI: 3.99–24.01, *p* < 0.001, Fig. 2). Median survival times for OS (PFS) were 164.3 months for the intermediate-risk group and 28.1 (23.0) months for the high-risk group. For the low-risk group (OS and PFS) and the intermediate-risk group (PFS) the median survival times could not be calculated (survival probability remained >0.5).

In the multivariate analysis (OS), which included age, sex, UICC stage, resection status, and TILs, patient age and the combined classification were identified as independent prognostic factors. However, for PFS, only the combined classification (TB and LVI) remained as an independent prognostic factor (Table 4).

**Table 4:** Results for the multivariate analysis.

			HR (mean)	HR (95% CI)	*p*
OS	Age		1.06	1.01–1.11	**0.010**
	Sex				
		male	1		
		female	0.98	0.37–2.62	0.974
	UICC stage				
		stage I/II	1		
		stage III	1.43	0.17–12.06	0.744
		stage IV	2.91	0.35–24.40	0.325
	Resection status				
		R0	1		
		R1/R2	2.16	0.75–6.26	0.156
		RX	0.79	0.19–3.19	0.738
	Combined classification (TB and LVI)				
		TB low and L0	1		
		TB low/L1, TB high/L0	3.30	1.11–9.77	**0.031**
		TB high and L1	13.58	3.83–48.11	**<0.001**
PFS	Age		1.02	0.97–1.08	0.409
	Sex				
		male	1		
		female	0.75	0.26–2.19	0.604
	UICC stage				
		stage I/II	1		
		stage III	0.84	0.17–4.20	0.827
		stage IV	1.21	0.23–6.34	0.823
	Resection status				
		R0	1		
		R1/R2	1.83	0.53–6.30	0.337
		RX	1.76	0.53–5.90	0.357
	Combined classification (TB and LVI)				
		TB low and L0	1		
		TB low/L1, TB high/L0	4.26	1.31–13.83	**0.016**
		TB high and L1	26.34	6.88–100.89	**<0.001**

*HR* Hazard ratio, *95% CI* 95% confidence interval, *OS* Overall survival, *PFS* Progression-free survival, *TB* Tumor budding (10 HPF), *TILs* Tumor infiltrating lymphocytes, *UICC* Union internationale contre le cancer.

Bold values indicate statistical significance.

For a subset of *n* = 115 cases (41.8%) the exact anatomic localization of the tumor was available. According to Tham et al. tumors located in the tonsils or base of tongue (*n* = 112) were considered tonsillar related areas whereas the tumors located at the lateral oropharyngeal wall and soft palate (*n* = 3) were considered to arise in a non-tonsillar related area [28].

Subsequently analyzing the combined stratification scheme (TB + LVI) with regards to tonsillar and non-tonsillar related areas and overall survival we observed that the combined scheme can prognostically stratify tumors of the tonsillar related areas. Here, in univariate analysis cases with high TB/absence of LVI or presence of LVI/low TB were allocated to the intermediate risk group (HR: 1.62, 95% CI: 0.33–8.04, *p* = 0.557). Cases with high TB and presence of LVI were allocated to the high-risk group (HR: 16.54, 95% CI: 2.64–103.66, *p* = 0.003). Among tonsillar related tumors similar results were obtained for cases with high TB and presence of LVI in multivariate analysis of overall survival (including age, sex, UICC stage, resection status and the combined scheme of TB and LVI) (HR: 29.00, 95% CI: 2.45–342.96, *p* = 0.008; Supplementary Table 5). Median survival times for tonsillar related and nontonsillar related areas are shown in Supplementary Table 6.

For non-tonsillar related areas no prognostic stratification considering the combined stratification scheme (TB + LVI) could be calculated (only *n* = 3 cases).

## Discussion

In 2017, the WHO classification of tumors of the head and neck separated HPV-associated OPSCC from HPV-independent counterparts [3] and this classification was adopted in the current WHO classification [24]. The rationale for this distinction is based on the evidence that these entities - HPV-positive versus HPV-negative OPSCC - exhibit differences in epidemiological, molecular, biological and prognostic characteristics [29–31]. Among HPV-positive OPSCC, a subgroup of approximately 20% shows a comparably poor prognosis linked to early cancer recurrences and metastasis [31, 32]. However, currently no established biomarker effectively distinguishes these high-risk cases from low-risk cases [31].

Aiming for improved patient stratification, we evaluated the feasibility of TB alongside other promising morphological parameters to identify low-risk and high-risk p16-positive OPSCC. Particularly in focus were our previously published TB grading scheme [18] as well as TIL. Furthermore, we evaluated TSR, which was shown to be a prognostic biomarker in HNSCC including OPSCC [19, 20]. After TB was identified as a tissue-based biomarker with significant prognostic value, we then aimed to improve the prognostic stratification by combining TB with LVI. The latter is a well-established biomarker which has been demonstrated to be of significant impact on survival. To this end we tested a combination of TB and LVI and were finally able to show that this combination revealed the highest prognostic significance, even stratifying the subgroup of tonsillar-related OPSCC.

Up to date only our own previous study analyzed TB in HPV-associated OPSCC [18]. In the respective study, we identified thresholds of 2 buds in 1 HPF and 6 buds in 10 HPF as prognostically most relevant. However, the study’s applicability on HPV-positive OPSCC was limited due to a relatively small number of patients with this type of cancer. The present study includes the largest number of p16-positive OPSCC patients to date. An extensive multicenter setting allowed us to reach this impressive number of patients which carries sufficient power for robust statistical analyses.

Applying the cutoffs 2 buds in 1 HPF and 6 buds in 10 HPF to our case series, we successfully stratified p16-positive OPSCC into low-risk and high-risk subgroups, observing significant differences in both OS and PFS in uni- and multivariate survival analysis for both, all OPSCC as well as the subgroup of OPSCC located within tonsillar related areas.

A topic of ongoing debate is the evaluation procedure of TB in HNSCC and squamous cell carcinomas in general. In colorectal cancer TB is evaluated in a 20x field (for further details see International Tumor Budding Consensus Conference [33]. In HNSCC (as well as in other squamous cell carcinoma entities including pulmonary squamous cell carcinoma), TB has been evaluated frequently in 1 HPF at 40x magnification (reviewed comprehensively in refs. [25, 34–36]). Consequently, we applied the latter HPF-method in our previous [18] as well as in our current study. To enable a transformation to digital evaluation, we calculated the size of 1 HPF in µm^2^, rendering an area of 97464 µm^2^being included in 1 HPF. Therefore, we believe that we are able to alleviate the comparability with other studies on TB in squamous cell carcinomas which preferably have applied this HPF method.

Cases with high TB were associated with poorer outcomes in univariate analysis and TB remained as an independent prognostic factor in multivariate analyses. Similar results were achieved when evaluating TB in both 1 and 10 HPF, indicating the applicability of TB assessment in both biopsy and resection specimens [37]. However, we recommend the 10 HPF method for evaluating TB if feasible as it has shown better reproducibility [33].

The negative prognostic impact of TB in p16-positive OPSCC aligns with similar findings in other cancer types, including HPV-negative HNSCC, colorectal cancer, lung cancer, breast cancer, and pancreatic cancer [25]. As numerous original studies and reviews have already demonstrated the prognostic value of TB in p16-negative HNSCC (reviewed amongst others in refs. [25, 35, 36, 38], we do not believe that an additional validation for this subtype is compatible with the design of our current study, which excludes p16-negative OPSCC. Furthermore, our results emphasize the prognostic value of TB in HNSCC: TB stands out as an easily applicable and cost-effective morphological parameter that may also be relevant for p16-positive OPSCC [39].

In our study, LVI emerged as another independent prognostic factor for worse outcomes regarding OS and PFS. Unlike TB, LVI is already considered an adverse factor in OPSCC, and its presence might lead to administering adjuvant radiotherapy [40, 41].

Taking into account that TB as well as LVI were significantly correlated with poor outcome, we probed to combine both in a combination scheme. Indeed, statistical analysis showed that the combination of TB and LVI is a significant prognosticator of patient outcome in uni- and multivariate analysis with even better capacity to stratify patients compared to each biomarker alone. Given the severe side effects associated with radio(chemo)therapy, several trials have explored de-intensification strategies in the treatment of p16-positive OPSCC [42]. From a clinical perspective, evaluating TB, particularly in conjunction with LVI, could help to distinguish low-risk p16-positive OPSCC patients who might benefit from less intensive treatment regimens from high-risk patients who may require the full spectrum of therapeutic options. The presence of TB, as a histomorphological indicator of aggressive tumor biology - particularly when co-occurring with LVI - may represent the morphological manifestation of a subset of p16-positive OPSCC associated with poor outcome. This subset shows comparably worse survival outcomes, despite the generally superior tumor biology characteristic of p16-positive OPSCC [25, 31, 43].

Tham et al. evaluated the influence of the anatomic subsite on survival in a large cohort of HPV-positive OPSCC and discriminated between tonsillar related areas and nontonsillar related areas. In our study the combined stratification scheme (TB and LVI) was able to identify a high-risk group of tonsillar related tumors (high TB and presence of LVI). However, no prognostic significance was reached for tumors with high TB and absence of LVI or low TB and presence of LVI. This might be attributable to the comparable small size of this subcohort with distinct anatomic subsites available. For nontonsillar related tumors no prognostic stratification of the combined scheme (TB and LVI) could be calculated as for this category only three cases could be assigned a distinct anatomic subsite.

Our results may pave the way to a patient stratification in analogy to colorectal cancer. Here, TB and LVI (amongst others) are combined in risk-scoring systems to identify patients with T1 carcinoma which need treatment escalation due to a high risk of metastasis and poor outcome [44]. This could be an approach for identification of high-risk cases amongst OPSCC patients as well.

Some authors have argued against conducting TB-scoring on HPV-associated HNSCC, positing that HPV-associated cancers typically do not exhibit TB, thus rendering TB scoring inappropriate [45]. However, in our analysis we included all p16-positive cases irrespective of their morphological characteristics. This approach is supported by literature suggesting that p16 is a reliable marker for an HPV-association in HNSCC located in the oropharynx [23, 24] and that there may be instances where HPV-associated cancers present with characteristics like a keratinizing phenotype [46, 47]. Although our analysis is not directly comparable to those involving molecularly confirmed HPV-positive OPSCC, our results nevertheless indicate a prognostically relevant stratification of p16-positive OPSCC by evaluating TB. Therefore, we believe that our study could provide relevant prognostic information for the treatment of patients with p16-positive OPSCC.

Interestingly, our study did not reveal an association between TB and the presence of extracapsular extension in lymph node metastasis. This lack of association might be due to the limited number of patients for whom data on extracapsular extension was available.

In our study, we did not observe any impact of TSR on the prognosis of p16-positive OPSCC. This finding is in contrast to a recent study by Almangush et al. [20] which demonstrated a poor outcome of HPV-associated OPSCC with high stromal content. However, most data on the prognostic impact of TSR in HNSCC is related to oral squamous cell carcinomas and data on the prognostic impact of TSR on other HNSCC subsites including the oropharynx is scarce [19, 21]. Taken together, the significance of TSR in OPSCC remains to be clarified in future studies.

However, our study has limitations. Current studies evaluating treatment de-escalation strategies for OPSCC typically consider factors such as an underlying HPV-association, tumor node metastasis (TNM) stage, and smoking status [48]. Due to the multicenter design of the study, additional molecular workups to confirm HPV-infection were not performed as access to formalin-fixed paraffin-embedded tumor tissue was not feasible; we only had access to digitized H&E-stained tissue sections from the five university centers. Likewise, our clinical data did not include information on patients’ smoking status or alcohol abuse. Thus, we were unable to directly assess whether TB/LVI is correlated with HPV-positivity based on RNA-or DNA-analysis, smoking status and/or alcohol abuse. One further drawback of our multicenter study design was that clinical data regarding the exact localization of the tumor within the oropharynx were not available for several cases. Thus, the prognostic significance of TB/LVI regarding nontonsillar related areas could not be determined. Therefore, additional studies specifically addressing these questions and examining if TB/LVI can provide valuable information in addition to the TNM stage in HPV-associated OPSCC are warranted.

Another limitation is the retrospective design of our study, with cases recruited over an extended period. This long timeframe may have led to variations in treatment courses due to changes in clinical practice. This limitation could be mitigated in future studies by evaluating TB/LVI in clinical trial populations treated more uniformly.

Nevertheless, we believe that our findings underscore the prognostic relevance of TB and LVI in p16-positive OPSCC and emphasize its potential utility in clinical practice.

## Conclusion

In our evaluation of previously described cutoffs for TB, we were able to stratify p16-positive OPSCC with significant prognostic implications. In addition to TB, LVI emerged as another independent prognostic factor associated with inferior outcomes. Thus, morphological parameters such as TB and LVI, particularly when combined, might help in guiding clinical decision making regarding the most appropriate treatment regimen, whether it be intensified or de-intensified, for patients with OPSCC.

## Supplementary information

Supplemental Material

STROBE checklist

## Data Availability

Data analyzed in this study can be requested by the corresponding authors.

## References

[1] Nogues JC, Fassas S, Mulcahy C, Zapanta PE. Human Papillomavirus-Associated Head and Neck Cancer. J Am Board Fam Med. 2021;34:832–7. 34312276 10.3122/jabfm.2021.04.200588

[2] Wang H, Zhang Y, Bai W, Wang B, Wei J, Ji R, et al. Feasibility of Immunohistochemical p16 Staining in the Diagnosis of Human Papillomavirus Infection in Patients With Squamous Cell Carcinoma of the Head and Neck: A Systematic Review and Meta-Analysis. Front Oncol. 2020;10:524928. 33324540 10.3389/fonc.2020.524928 PMC7724109

[3] El-Naggar AK, Chan JKC, Grandis JR, Slootweg PJ. WHO Classification of Head and Neck Tumours. Lyon (France): IARC Who Classification of Tum; 2017. 347.

[4] Sedghizadeh PP, Billington WD, Paxton D, Ebeed R, Mahabady S, Clark GT, et al. Is p16-positive oropharyngeal squamous cell carcinoma associated with favorable prognosis? A systematic review and meta-analysis. Oral Oncol. 2016;54:15–27. 26794879 10.1016/j.oraloncology.2016.01.002

[5] Bol V, Grégoire V. Biological basis for increased sensitivity to radiation therapy in HPV-positive head and neck cancers. Biomed Res Int. 2014;2014:696028. 24804233 10.1155/2014/696028 PMC3996288

[6] Rosenthal DI, Harari PM, Giralt J, Bell D, Raben D, Liu J, et al. Association of Human Papillomavirus and p16 Status With Outcomes in the IMCL-9815 Phase III Registration Trial for Patients With Locoregionally Advanced Oropharyngeal Squamous Cell Carcinoma of the Head and Neck Treated With Radiotherapy With or Without Cetuximab. J Clin Oncol. 2016;34:1300–8. 26712222 10.1200/JCO.2015.62.5970 PMC5070577

[7] Rahimi S. HPV-related squamous cell carcinoma of oropharynx: a review. J Clin Pathol. 2020;73:624–9. 32499224 10.1136/jclinpath-2020-206686

[8] Brierley JD, Gospodarowicz MK, Wittekind C. TNM Classification of Malignant Tumours. Hoboken, New Jersey (USA): John Wiley & Sons; 2017. 272 p.

[9] Cancer Genome Atlas Network. Comprehensive genomic characterization of head and neck squamous cell carcinomas. Nature. 2015;517:576–82. 25631445 10.1038/nature14129 PMC4311405

[10] Strohl MP, Wai KC, Ha PK. De-intensification strategies in HPV-related oropharyngeal squamous cell carcinoma-a narrative review. Ann Transl Med. 2020;8:1601. 33437800 10.21037/atm-20-2984 PMC7791209

[11] Brook I. Early side effects of radiation treatment for head and neck cancer. Cancer Radiother. 2021;25:507–13. 33685809 10.1016/j.canrad.2021.02.001

[12] Golusinski P, Corry J, Poorten VV, Simo R, Sjögren E, Mäkitie A, et al. De-escalation studies in HPV-positive oropharyngeal cancer: How should we proceed? Oral Oncol. 2021;123:105620. 34798575 10.1016/j.oraloncology.2021.105620

[13] Mirghani H, Blanchard P. Treatment de-escalation for HPV-driven oropharyngeal cancer: Where do we stand? Clin Transl Radiat Oncol. 2018;8:4–11. 29594236 10.1016/j.ctro.2017.10.005 PMC5862680

[14] Mäkitie AA, Almangush A, Rodrigo JP, Ferlito A, Leivo I. Hallmarks of cancer: Tumor budding as a sign of invasion and metastasis in head and neck cancer. Head Neck. 2019;41:3712–8. 31328847 10.1002/hed.25872

[15] Boxberg M, Bollwein C, Jöhrens K, Kuhn PH, Haller B, Steiger K, et al. Novel prognostic histopathological grading system in oral squamous cell carcinoma based on tumour budding and cell nest size shows high interobserver and intraobserver concordance. J Clin Pathol. 2019;72:285–94. 30530818 10.1136/jclinpath-2018-205454

[16] Jesinghaus M, Steiger K, Stögbauer F, Haller B, Kolk A, Straßen U, et al. Pre-operative cellular dissociation grading in biopsies is highly predictive of post-operative tumour stage and patient outcome in head and neck squamous cell carcinoma. Br J Cancer. 2020;122:835–46. 31937923 10.1038/s41416-019-0719-8 PMC7078181

[17] Okuyama K, Suzuki K, Yanamoto S. Relationship between Tumor Budding and Partial Epithelial-Mesenchymal Transition in Head and Neck Cancer. Cancers. 2023;15. Available from: 10.3390/cancers15041111. 10.3390/cancers15041111 PMC9953904 36831453

[18] Stögbauer F, Beck S, Ourailidis I, Hess J, Poremba C, Lauterbach M, et al. Tumour budding-based grading as independent prognostic biomarker in HPV-positive and HPV-negative head and neck cancer. Br J Cancer. 2023;128:2295–306. 37045906 10.1038/s41416-023-02240-y PMC10241901

[19] Almangush A, Alabi RO, Troiano G, Coletta RD, Salo T, Pirinen M, et al. Clinical significance of tumor-stroma ratio in head and neck cancer: a systematic review and meta-analysis. BMC Cancer. 2021;21:480. 33931044 10.1186/s12885-021-08222-8 PMC8086072

[20] Almangush A, Jouhi L, Haglund C, Hagström J, Mäkitie AA, Leivo I. Tumor-stroma ratio is a promising prognostic classifier in oropharyngeal cancer. Hum Pathol. 2023;136:16–24. 37001738 10.1016/j.humpath.2023.03.010

[21] Morais EF, Morais HG, Martins HD, Carlan LM, Costa AD, Freitas RD. Prognostic and clinicopathological significance of tumor-stroma ratio in head and neck squamous cell carcinoma: A systematic review. Med Oral Patol Oral Cir Bucal. 2022;27:e301–9. 35717622 10.4317/medoral.24922 PMC9271351

[22] Almangush A, Jouhi L, Atula T, Haglund C, Mäkitie AA, Hagström J, et al. Tumour-infiltrating lymphocytes in oropharyngeal cancer: a validation study according to the criteria of the International Immuno-Oncology Biomarker Working Group. Br J Cancer. 2022;126:1589–94. 35043007 10.1038/s41416-022-01708-7 PMC9130301

[23] Lewis JS Jr, Beadle B, Bishop JA, Chernock RD, Colasacco C, Lacchetti C, et al. Human Papillomavirus Testing in Head and Neck Carcinomas: Guideline From the College of American Pathologists. Arch Pathol Lab Med. 2018;142:559–97. 29251996 10.5858/arpa.2017-0286-CP

[24] WHO Classification of Tumours Editorial Board. Head and Neck Tumours: Who Classification of Tumours. Lyon (France): World Health Organization; 2024. 836.

[25] Lugli A, Zlobec I, Berger MD, Kirsch R, Nagtegaal ID. Tumour budding in solid cancers. Nat Rev Clin Oncol. 2021;18:101–15. 32901132 10.1038/s41571-020-0422-y

[26] Hendry S, Salgado R, Gevaert T, Russell PA, John T, Thapa B, et al. Assessing Tumor-Infiltrating Lymphocytes in Solid Tumors: A Practical Review for Pathologists and Proposal for a Standardized Method from the International Immuno-Oncology Biomarkers Working Group: Part 2: TILs in Melanoma, Gastrointestinal Tract Carcinomas, Non-Small Cell Lung Carcinoma and Mesothelioma, Endometrial and Ovarian Carcinomas, Squamous Cell Carcinoma of the Head and Neck, Genitourinary Carcinomas, and Primary Brain Tumors. Adv Anat Pathol. 2017;24:311–35. 28777143 10.1097/PAP.0000000000000161 PMC5638696

[27] Budczies J, Klauschen F, Sinn BV, Győrffy B, Schmitt WD, Darb-Esfahani S, et al. Cutoff Finder: a comprehensive and straightforward Web application enabling rapid biomarker cutoff optimization. PLoS One. 2012;7:e51862. 23251644 10.1371/journal.pone.0051862 PMC3522617

[28] Tham T, Ahn S, Frank D, Kraus D, Costantino P. Anatomical subsite modifies survival in oropharyngeal squamous cell carcinoma: National Cancer Database study. Head Neck. 2020;42:434–45. 31773842 10.1002/hed.26019

[29] Boscolo-Rizzo P, Pawlita M, Holzinger D. From HPV-positive towards HPV-driven oropharyngeal squamous cell carcinomas. Cancer Treat Rev. 2016;42:24–9. 26547133 10.1016/j.ctrv.2015.10.009

[30] Chen AM. De-escalated radiation for human papillomavirus virus-related oropharyngeal cancer: Who, why, what, where, when, how, how much and what next? Radiother Oncol. 2024;8;110373. 10.1016/j.radonc.2024.110373 38857702

[31] Lechner M, Liu J, Masterson L, Fenton TR. HPV-associated oropharyngeal cancer: epidemiology, molecular biology and clinical management. Nat Rev Clin Oncol. 2022;19:306–27. 35105976 10.1038/s41571-022-00603-7 PMC8805140

[32] Huang SH, Perez-Ordonez B, Weinreb I, Hope A, Massey C, Waldron JN, et al. Natural course of distant metastases following radiotherapy or chemoradiotherapy in HPV-related oropharyngeal cancer. Oral Oncol. 2013;49:79–85. 22917550 10.1016/j.oraloncology.2012.07.015

[33] Lugli A, Kirsch R, Ajioka Y, Bosman F, Cathomas G, Dawson H, et al. Recommendations for reporting tumor budding in colorectal cancer based on the International Tumor Budding Consensus Conference (ITBCC) 2016. Mod Pathol. 2017;30:1299–311. 28548122 10.1038/modpathol.2017.46

[34] Wankhede D, Hofman P, Grover S. Prognostic impact of tumour budding in squamous cell carcinoma of the lung: a systematic review and meta-analysis. Histopathology. 2023;82:521–30. 36217904 10.1111/his.14822

[35] Almangush A, Mäkitie AA, Leivo I. Tumour budding in head and neck cancer: what have we learnt and the next steps towards clinical implementation. Br J Cancer. 2024;130:1–2. 38097743 10.1038/s41416-023-02531-4 PMC10781682

[36] Zanoletti E, Daloiso A, Nicolè L, Cazzador D, Mondello T, Franz L, et al. Tumor budding to investigate local invasion, metastasis, and prognosis of head and neck carcinoma: A systematic review. Head Neck. 2024;46:651–71. 38013617 10.1002/hed.27583

[37] Wen X, Zee SY, Shroyer KR, Bandovic J. Intratumoral Budding and Tumor Microenvironment in Pretreatment Rectal Cancer Biopsies Predict the Response to Neoadjuvant Chemoradiotherapy. Appl Immunohistochem Mol Morphol. 2022;30:1–7. 34369419 10.1097/PAI.0000000000000966

[38] Almangush A, Pirinen M, Heikkinen I, Mäkitie AA, Salo T, Leivo I. Tumour budding in oral squamous cell carcinoma: a meta-analysis. Br J Cancer. 2018;118:577–86. 29190636 10.1038/bjc.2017.425 PMC5830589

[39] Gupta S, Sreeram S, Pinto AC, Suresh PK, Basavaiah SH. Tumor Budding Assessment with Cytokeratin and Its Significance in Laryngeal Squamous Cell Carcinomas. Indian J Otolaryngol Head Neck Surg. 2022;74:494–500. 36514426 10.1007/s12070-021-02981-3 PMC9741683

[40] Park YM, Lim JY, Koh YW, Choi EC, Kim SH. Surgical margin status and role of adjuvant therapy in human papillomavirus-positive oropharyngeal cancer. Head Neck. 2023;45:2369–76. 37489048 10.1002/hed.27473

[41] Quon H, Vapiwala N, Forastiere A, Kennedy EB. Radiation Therapy for Oropharyngeal Squamous Cell Carcinoma: American Society of Clinical Oncology Endorsement of the American Society for Radiation Oncology Evidence-Based Clinical Practice Guideline Summary. J Oncol Pr. 2018;14:117–22. 10.1200/JOP.2017.025247 29068751

[42] Tawk B, Debus J, Abdollahi A. Evolution of a Paradigm Switch in Diagnosis and Treatment of HPV-Driven Head and Neck Cancer-Striking the Balance Between Toxicity and Cure. Front Pharm. 2021;12:753387. 10.3389/fphar.2021.753387 PMC8810823 35126105

[43] Alessandrini L, Zanoletti E, Cazzador D, Sbaraglia M, Franz L, Tealdo G, et al. Tumor budding to investigate local invasion, metastasis and prognosis in temporal bone squamous cell carcinoma. Pathol Res Pr. 2022;229:153719. 10.1016/j.prp.2021.153719 34953406

[44] Ichimasa K, Kudo SE, Miyachi H, Kouyama Y, Misawa M, Mori Y. Risk Stratification of T1 Colorectal Cancer Metastasis to Lymph Nodes: Current Status and Perspective. Gut Liver. 2021;15:818–26. 33361548 10.5009/gnl20224 PMC8593512

[45] Chiesa-Estomba CM, Thompson L, Agaimy A, Zidar N, Simpson RHW, Franchi A, et al. Predictive value of tumor budding in head and neck squamous cell carcinoma: an update. Virchows Arch. 2023; Available from: 10.1007/s00428-023-03630-6. 10.1007/s00428-023-03630-6 37642731

[46] Molony P, Werner R, Martin C, Callanan D, Nauta I, Heideman D, et al. The role of tumour morphology in assigning HPV status in oropharyngeal squamous cell carcinoma. Oral Oncol. 2020;105:104670. 32279011 10.1016/j.oraloncology.2020.104670

[47] Mehanna H, Taberna M, von Buchwald C, Tous S, Brooks J, Mena M, et al. Prognostic implications of p16 and HPV discordance in oropharyngeal cancer (HNCIG-EPIC-OPC): a multicentre, multinational, individual patient data analysis. Lancet Oncol. 2023;24:239–51. 36796393 10.1016/S1470-2045(23)00013-X

[48] Economopoulou P, Kotsantis I, Psyrri A. De-Escalating Strategies in HPV-Associated Head and Neck Squamous Cell Carcinoma. Viruses. 2021;13. Available from: 10.3390/v13091787. 10.3390/v13091787 PMC8473011 34578368

