# Supplementary material for: Tumor budding and lymphovascular invasion as prognostic factors in p16-positive oropharyngeal squamous cell carcinomas
Source: Br J Cancer. 2024 Nov 29;132(2):212–21. doi: 10.1038/s41416-024-02912-3 (PMC11747400; doi:10.1038/s41416-024-02912-3)
Supplement: Supplementary file 1 — Supplemental Material [file 41416_2024_2912_MOESM1_ESM.docx]

# Supplemental material

## **Supplemental Table 1:** Clinicopathological characteristics of the cohort. IQR: Interquartile range, N/A: Not available.

|  | n | % |
| --- | --- | --- |
| **Age** | median 61 years (IQR 13 years) | |
| **Sex** |  |  |
| male | 192 | 69.9 |
| female | 83 | 30.1 |
| **Anatomic subsite in the oropharynx** |  |  |
| Tonsils | 95 | 34.5 |
| Base of tongue | 17 | 6.2 |
| Lateral oropharyngeal wall | 2 | 0.7 |
| Soft palate | 1 | 0.4 |
| Oropharynx (no specific anatomic subsite) | 160 | 58.2 |
| **pT** |  |  |
| pT1 | 78 | 30.7 |
| pT2 | 136 | 50.9 |
| pT3 | 35 | 13.4 |
| pT4 | 11 | 4.9 |
| N/A | 15 |  |
| **pN** |  |  |
| pN0 | 54 | 20.3 |
| pN+ | 194 | 79.7 |
| N/A | 27 |  |
| **UICC stage** |  |  |
| I/II | 48 | 17.6 |
| III | 117 | 48.0 |
| IV | 92 | 34.4 |
| N/A | 18 |  |
| **Resection status** |  |  |
| R0 | 160 | 78.2 |
| R1/R2 | 30 | 13.9 |
| RX | 17 | 7.9 |
| N/A | 68 |  |
| **Extracapsular extension** |  |  |
| absent | 45 | 71.6 |
| present | 19 | 28.4 |
| N/A | 211 |  |
| **Lymphovascular invasion** |  |  |
| absent | 224 | 81.5 |
| present | 51 | 18.5 |
| **Perineural invasion** |  |  |
| absent | 240 | 87.3 |
| present | 35 | 12.7 |
| **Morphology** |  |  |
| basaloid | 82 | 29.8 |
| conventional (keratinizing) | 78 | 28.4 |
| conventional (non-keratinizing) | 115 | 41.8 |
| **Center** |  |  |
| Erlangen | 128 | 42.8 |
| Heidelberg | 36 | 12.0 |
| Kiel | 16 | 5.4 |
| Lübeck | 68 | 22.7 |
| Munich | 51 | 17.1 |

## **Supplemental Table 2:** Mean, Median, minimum values and maximum values for TB in 1 HPF, TB in 10 HPF, stroma content and TILs. HPF: High-power field, TB: Tumor budding, TIL: Tumor infiltrating lymphocyte

##

|  | **Mean** | **Median** | **Minimum** | **Maximum** |
| --- | --- | --- | --- | --- |
| **TB in 1 HPF** | 1 | 1 | 0 | 13 |
| **TB in 10 HPF** | 2 | 1 | 0 | 44 |
| **Stroma content** | 34 | 40 | 4 | 75 |
| **TILs** | 39 | 30 | 1 | 90 |

##

##

##

##

## **Supplemental Table 3:** Results for the univariate analysis are shown for TB assessment in 10 HPF and for progression-free survival. HR: Hazard ratio, 95% CI: 95% confidence interval, TIL: Tumor infiltrating lymphocyte, UICC: Union internationale contre le cancer

|  |  | HR | HR (95% CI) | p |
| --- | --- | --- | --- | --- |
| **Age** |  | 1.03 | 1.00-1.07 | 0.084 |
| **Year of surgery** |  | 0.98 | 0.94-1.04 | 0.554 |
| **Sex** |  |  |  |  |
|  | male | 1 |  |  |
|  | female | 0.60 | 0.28-1.29 | 0.191 |
| **UICC stage** |  |  |  |  |
|  | stage I/II | 1 |  |  |
|  | stage III | 1.20 | 0.39-3.66 | 0.746 |
|  | stage IV | 2.15 | 0.71-6.47 | 0.175 |
| **Resection status** |  |  |  |  |
|  | R0 | 1 |  |  |
|  | R1/R2 | 2.18 | 0.85-5.65 | 0.107 |
|  | RX | 2.65 | 0.97-7.28 | 0.058 |
| **Lymphovascular invasion** |  |  |  |  |
|  | absent | 1 |  |  |
|  | present | 2.99 | 1.51-5.92 | **0.002** |
| **Perineural invasion** |  |  |  |  |
|  | absent | 1 |  |  |
|  | present | 1.45 | 0.60-3.49 | 0.407 |
| **Morphology** |  |  |  |  |
|  | basaloid | 1 |  |  |
|  | keratinizing | 1.02 | 0.40-2.61 | 0.960 |
|  | non-keratinizing | 1.29 | 0.56-2.99 | 0.545 |
| **Center** |  |  |  |  |
|  | Erlangen | 1 |  |  |
|  | Heidelberg | 0.95 | 0.39-2.35 | 0.915 |
|  | Kiel | 0 | 0-Inf | 0.997 |
|  | Lübeck | 0.86 | 0.39-1.90 | 0.706 |
|  | Munich | 0.60 | 0.20-1.78 | 0.357 |
| **TIL** |  |  |  |  |
|  | low | 1 |  |  |
|  | high | 0.75 | 0.36-1.57 | 0.443 |
| **Tumor budding** |  |  |  |  |
|  | low | 1 |  |  |
|  | high | 2.89 | 1.49-5.58 | **0.002** |

##

## **Supplemental Table 4:** Results for the multivariate analysis are shown for TB assessment in 10 HPF and for progression-free survival. HPF: high-power field, LVI: lymphovascular invasion, TB: tumor budding, UICC: Union internationale contre le cancer

|  |  | HR (mean) | HR (95% CI) | p |
| --- | --- | --- | --- | --- |
| Age |  | 1.02 | 0.97-1.07 | 0.405 |
| Sex |  |  |  |  |
|  | male | 1 |  |  |
|  | female | 0.78 | 0.27-2.30 | 0.658 |
| UICC stage |  |  |  |  |
|  | stage I/II | 1 |  |  |
|  | stage III | 0.65 | 0.12-3.60 | 0.624 |
|  | stage IV | 1.00 | 0.18-5.50 | 0.999 |
| Resection status |  |  |  |  |
|  | R0 | 1 |  |  |
|  | R1/R2 | 1.95 | 0.58-6.52 | 0.277 |
|  | RX | 2.17 | 0.60-7.84 | .0238 |
| Lymphovascular invasion |  |  |  |  |
|  | absent | 1 |  |  |
|  | present | 5.76 | 2.16-15.37 | **<0.001** |
| TILs |  |  |  |  |
|  | absent | 1 |  |  |
|  | present | 1.85 | 0.65-5.21 | 0.247 |
| Tumor budding |  |  |  |  |
|  | low | 1 |  |  |
|  | high | 5.97 | 2.32-15.41 | **<0.001** |

## **Supplemental Table 5:** Results for the multivariate analysis for the subgroup of tonsillar related tumors are shown for TB assessment in 10 HPF and for overall survival. HPF: high-power field, LVI: lymphovascular invasion, TB: tumor budding, UICC: Union internationale contre le cancer

|  |  | HR (mean) | HR (95% CI) | p |
| --- | --- | --- | --- | --- |
| Age |  | 1.10 | 0.99-1.21 | 0.053 |
| Sex |  |  |  |  |
|  | male | 1 |  |  |
|  | female | 1.33 | 0.15-12.13 | 0.802 |
| UICC stage |  |  |  |  |
|  | stage I/II | 1 |  |  |
|  | stage III | 1.37 | 0.00-Inf | 0.999 |
|  | stage IV | 5.64e+07 | 0.00-Inf | 0.999 |
| Resection status |  |  |  |  |
|  | R0 | 1 |  |  |
|  | R1/R2 | 1.67 | 1.23-226.77 | **0.034** |
|  | RX | 1.56 | 0.07-32.97 | 0.776 |
| Combined classification (TB and LVI) |  |  |  |  |
|  | Low TB+LVI absent | 1 |  |  |
|  | High TB+LVI absent/low TB+LVI present  High TB+LVI present | 1.06  2.90 | 0.14-7.83  2.45-342.96 | 0.952  **0.008** |

**Supplemental Table 6:** Median survival times for OS and PFS for tonsillar related areas.

|  | **OS (months)** | **PFS (months)** |
| --- | --- | --- |
| **Tonsillar related areas** |  |  |
| Low TB+LVI absent | N/A | N/A |
| High TB+LVI absent/low TB+LVI present | N/A | N/A |
| High TB+LVI present | 8.5 | 23.0 |

##
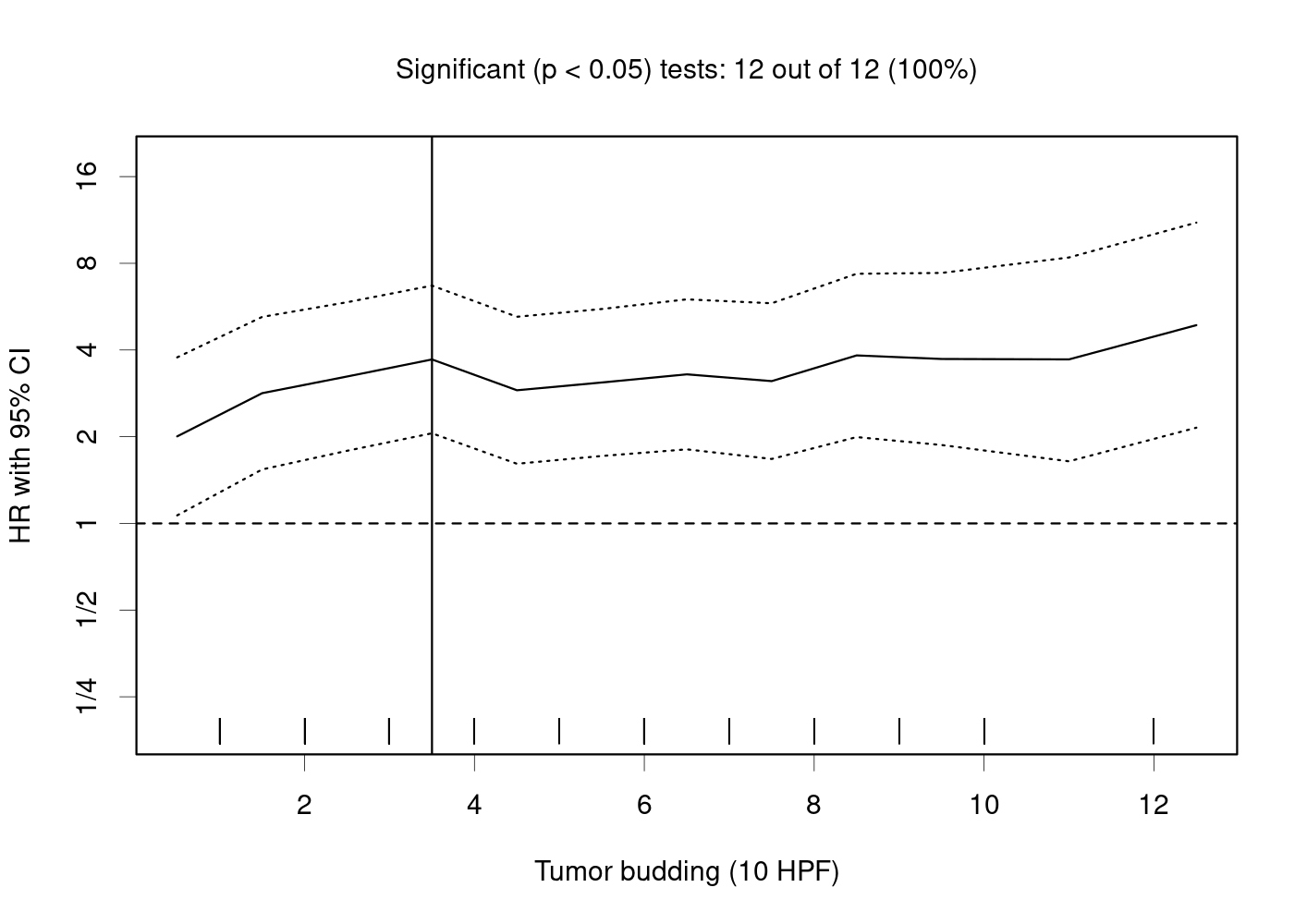


## **Supplemental Figure 1:** Hazard ratios and 95% confidence intervals are shown applying different cutoffs from 1 to 12. For all cutoffs the mean hazard ratios and the 95% confidence intervals are above 1 but the cutoff yielding the lowest p-value is ≥ 4 buds for TB high. 95% CI: 95% confidence interval, HPF: high-power field HR: hazard ratio, TB: tumor budding.

##

##

##

A) B)


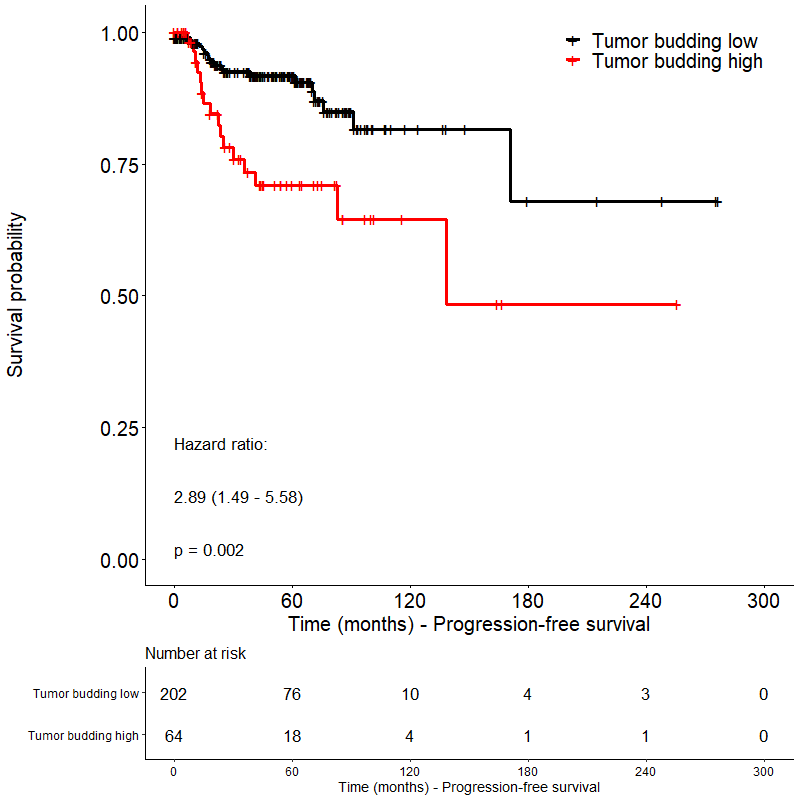


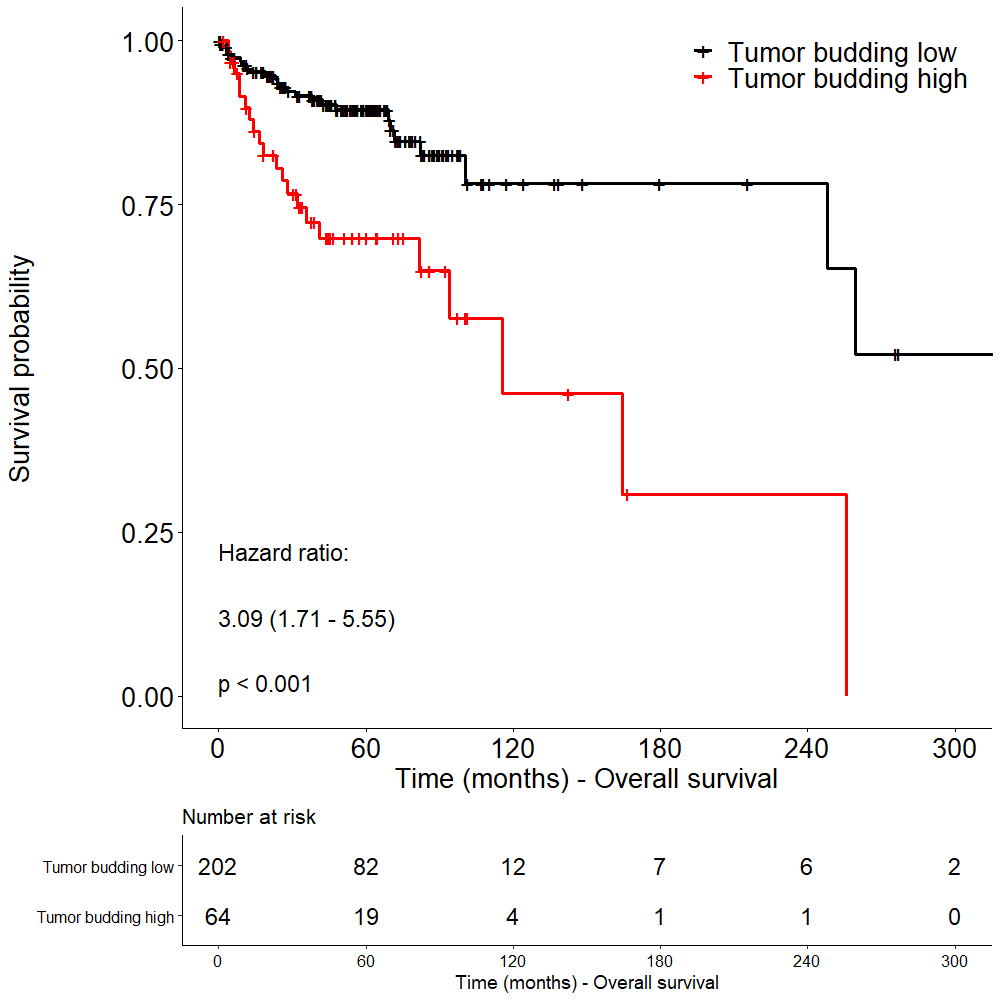


C) D)


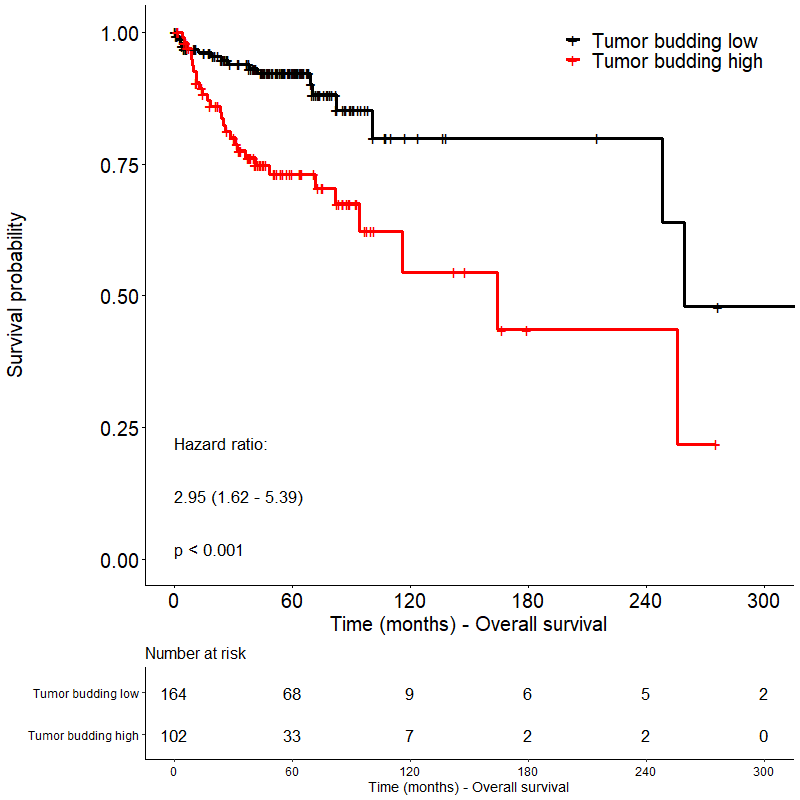

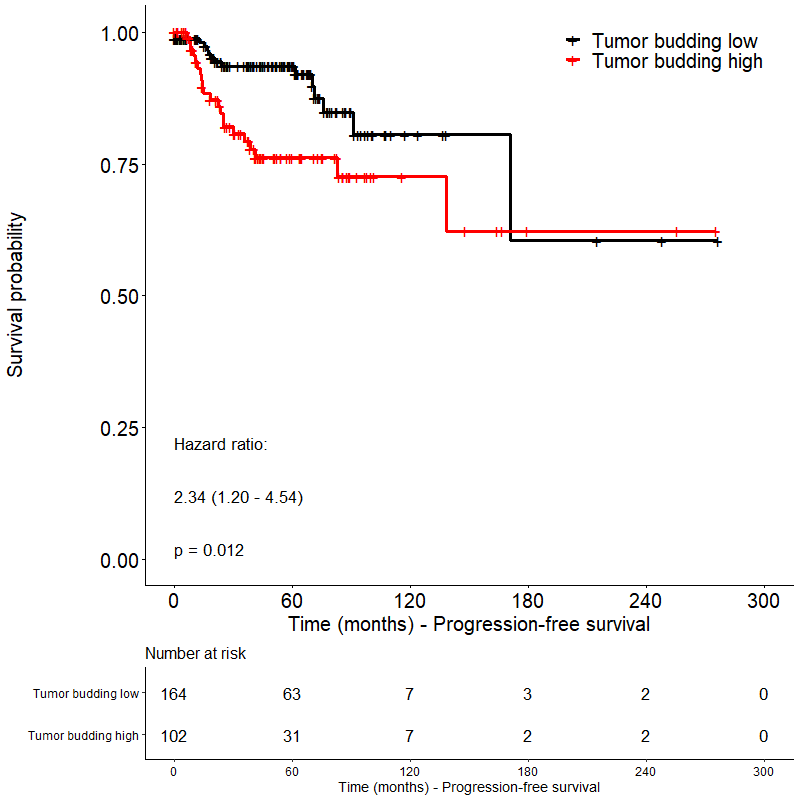


## **Supplemental Figure 2:** Kaplan-Meier plots for overall and progression-free survival. Patients were stratified into TB high and TB low according for TB in 10 HPF (A, B) and in 1 HPF (C, D). HPF: high-power field, TB: tumor budding.

A) B)


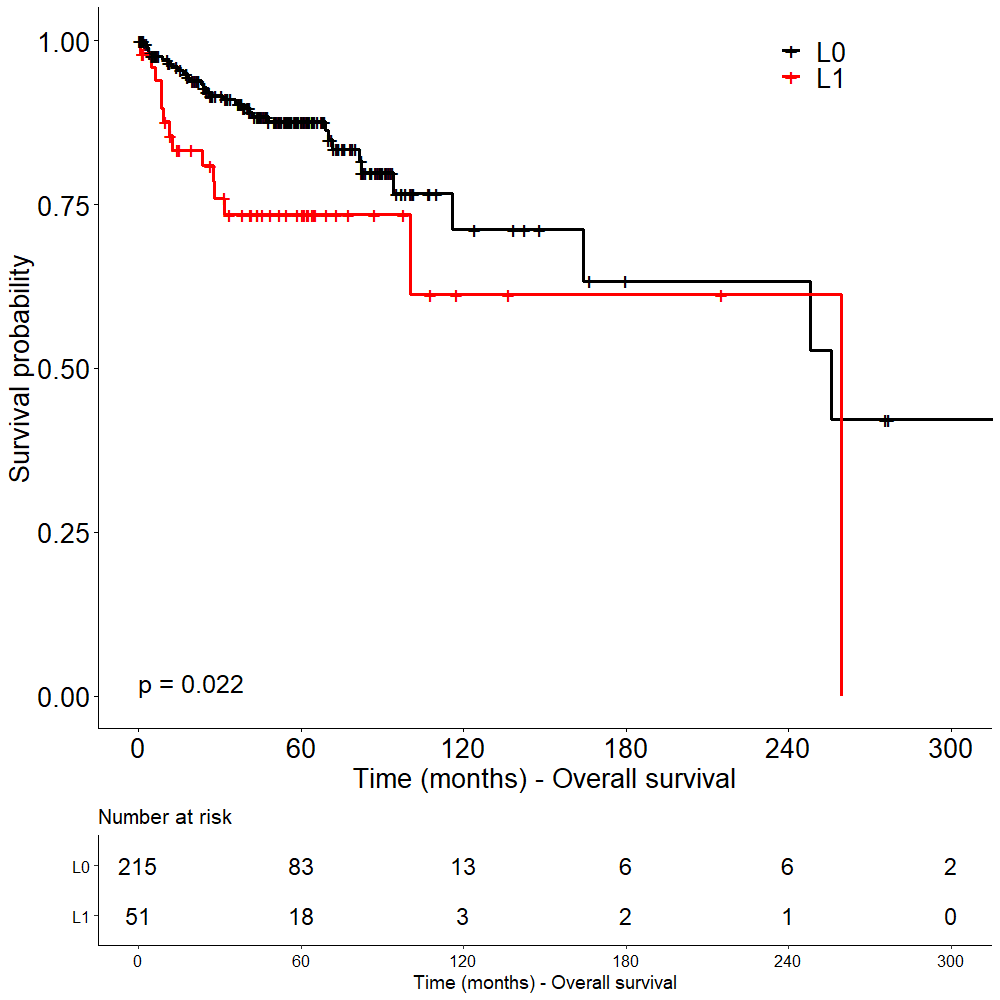

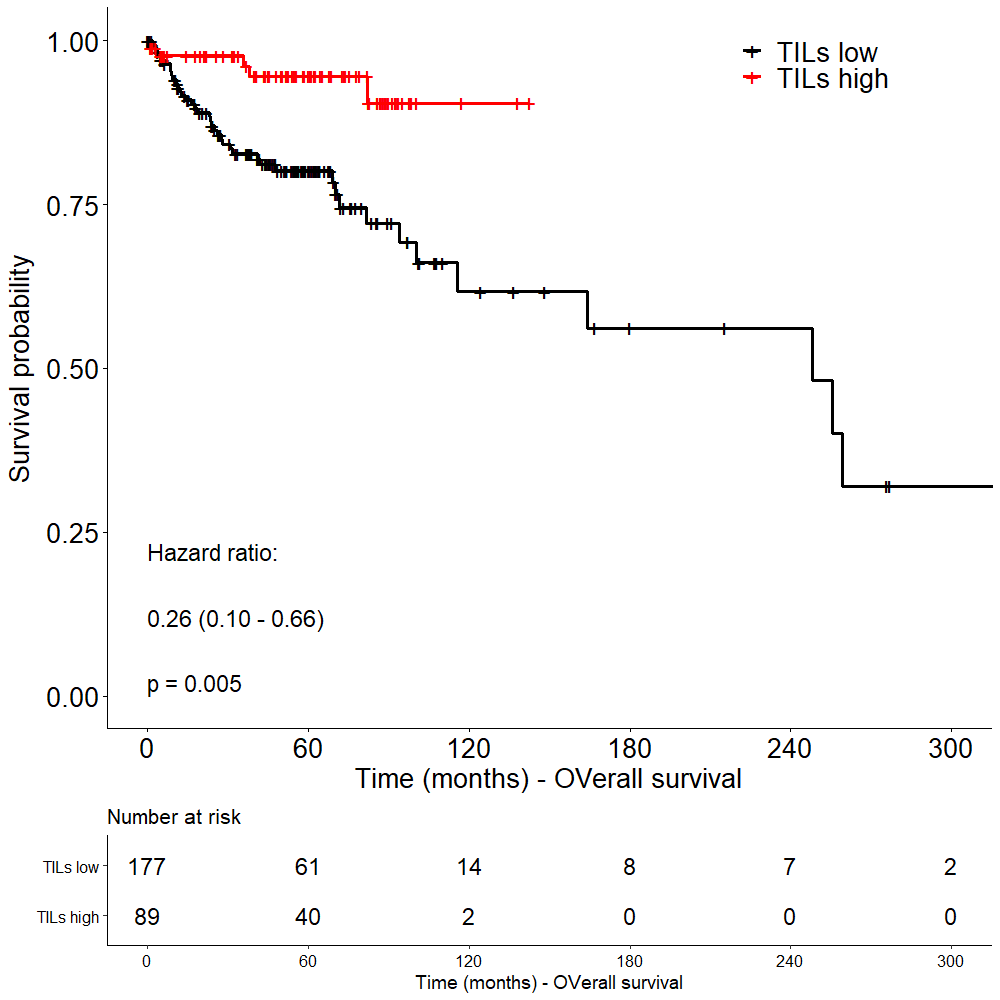

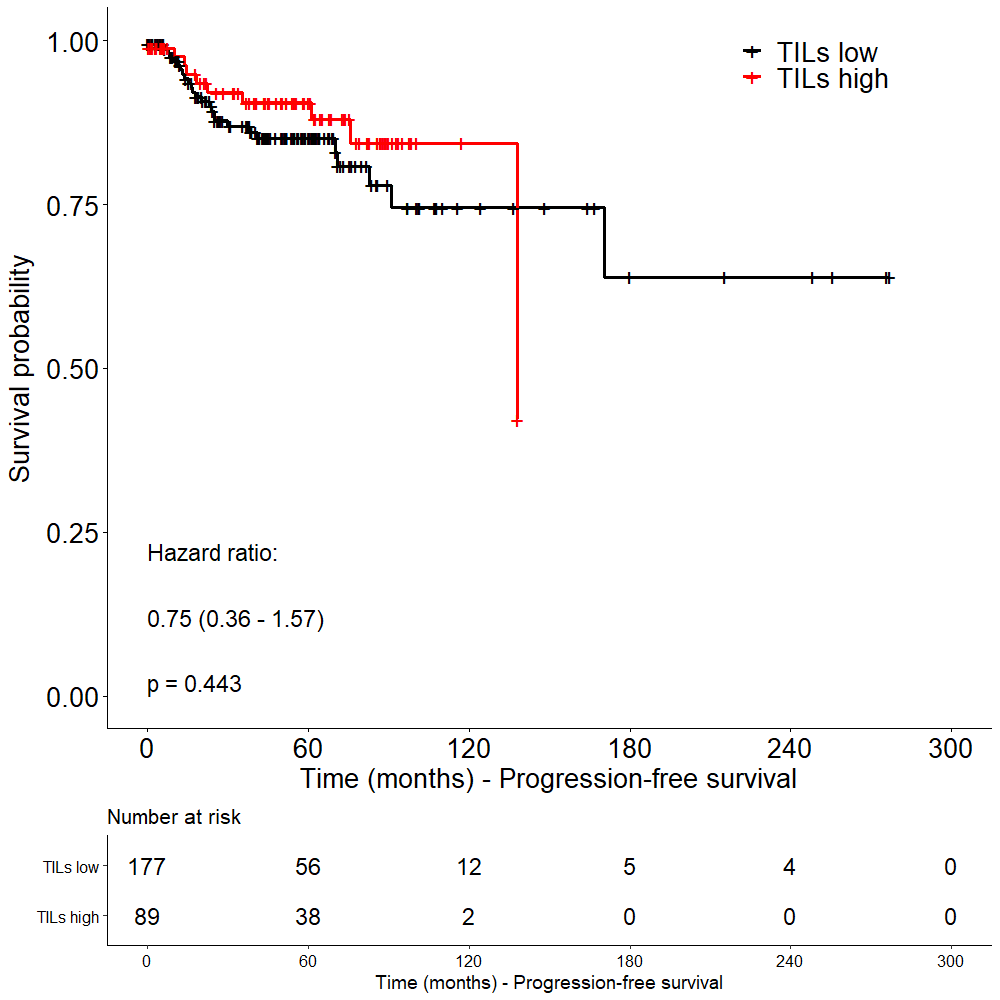


C) D)

**
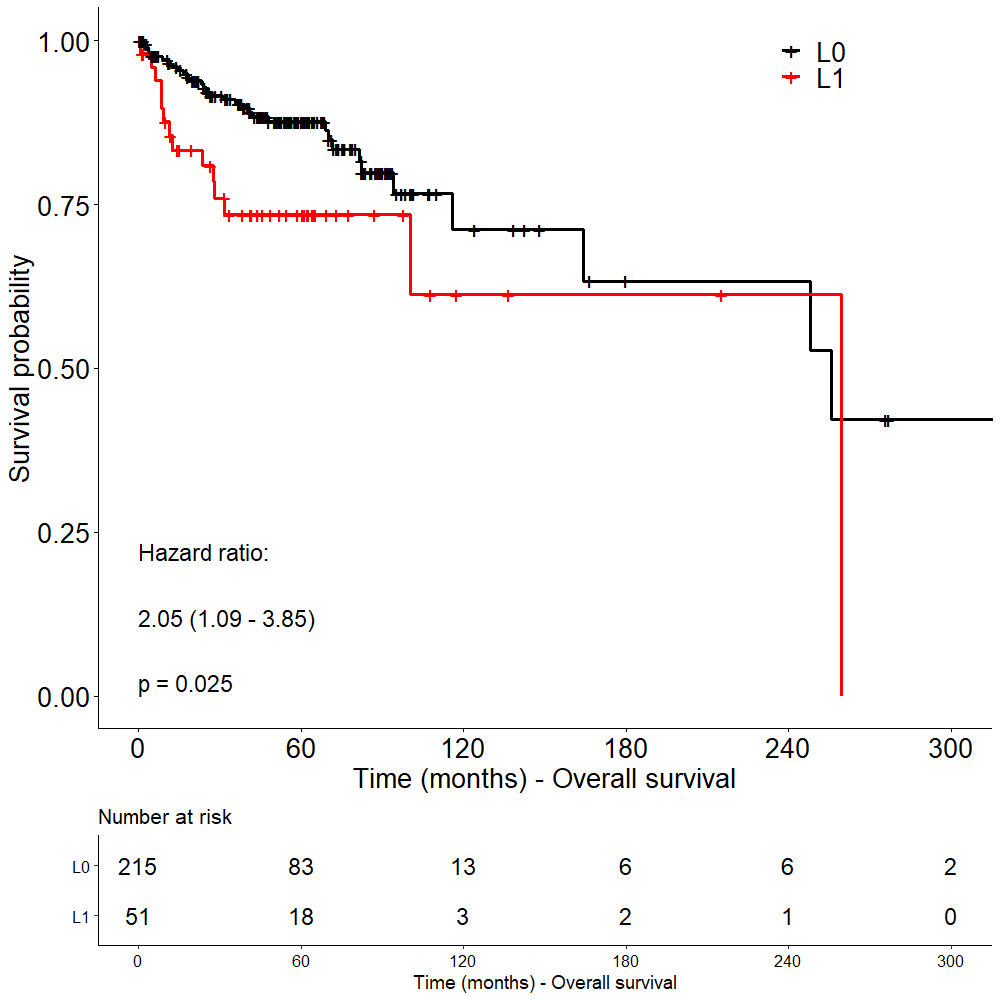

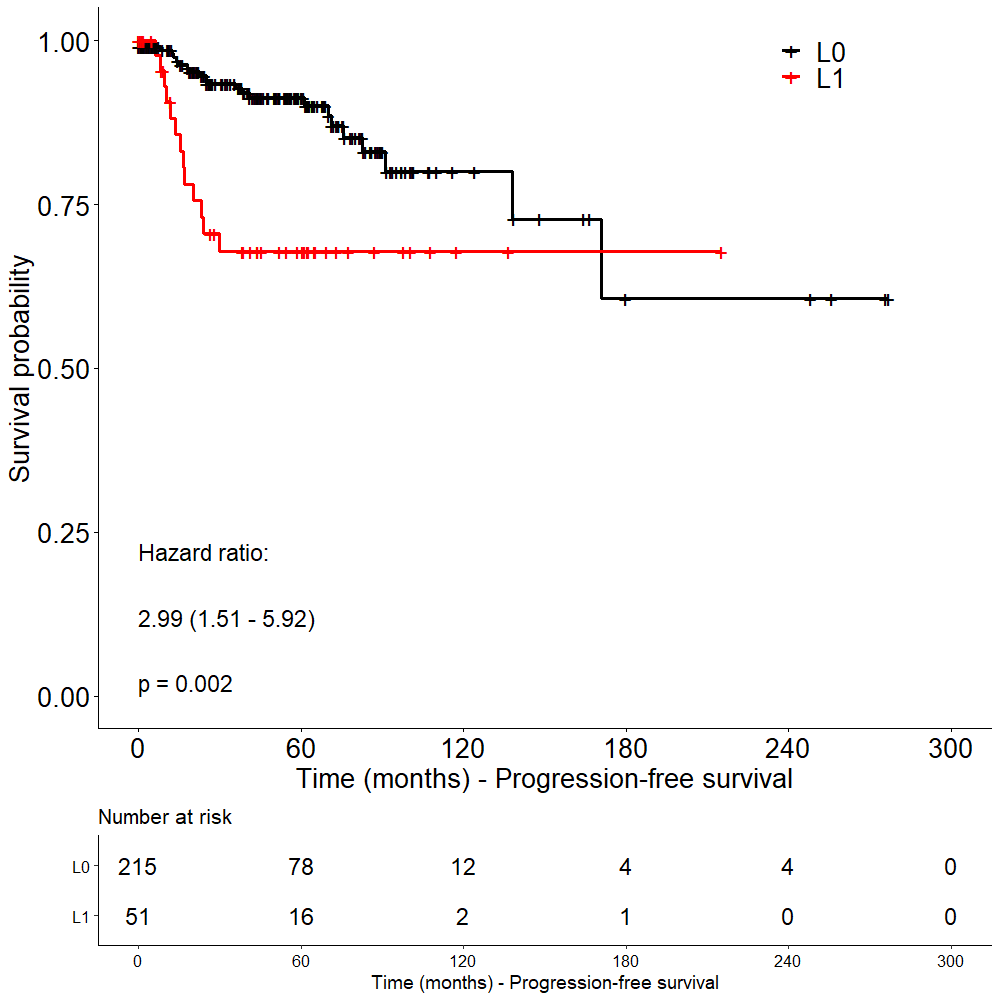
**

**Supplemental Figure 3:** Kaplan-Meier plots for overall and progression-free survival. Patients were stratified into TILs low and high (A, B) and LVI absent and present (C,D). LVI: lymphovascular invasion, TILs: tumor infiltrating lymphocytes.
